# Bridging the environment and neurodevelopment for children’s health: Associations between real-time air pollutant exposures and cognitive outcomes

**DOI:** 10.3389/fpsyg.2022.933327

**Published:** 2022-10-18

**Authors:** Josh Medrano, Natalie Crnosija, Richard W. Prather, Devon Payne-Sturges

**Affiliations:** ^1^Department of Human Development and Quantitative Methodology, University of Maryland, College Park, MD, United States; ^2^Maryland Institute for Applied Environmental Health, School of Public Health, University of Maryland, College Park, MD, United States

**Keywords:** air pollution, PM_2.5_, executive function, middle childhood, mathematics

## Abstract

Research suggests that children’s exposure to pollutants may impact their neurocognitive development. While researchers have found associations between air pollutants and cognitive development, these associations remain underspecified. Further, these exposures occur in the context of the built environment and may be exacerbated by local social vulnerability; in this context, individuals may experience a suite of socioenvironmental stressors that lead to increased cumulative risk exposure. In this pilot study, we tested whether real-time-measured personal exposure to PM_2.5_ relates to children’s executive function and mathematical skills, outcomes that may predict later mathematical performance, general academic performance and even employment outcomes. We recruited 30 families to participate in two rounds in Winter 2020 and Summer 2021. We collected children’s demographic data, as well as data about their living environment. In each round, children carried a small device that collected real-time ambient air pollution data for 3 days; parents logged their children’s activities each day. On the last day, children completed cognitive assessments indexing their working memory (n-back), inhibitory control (Go/No-Go), nonsymbolic math skills (dot comparison), and arithmetic skills (equation verification). Overall, 29 participants had pollutant readings from both rounds, and 21 had a full dataset. Nonparametric statistical analysis revealed no significant differences in ambient air pollution and cognitive performance over time, Spearman’s rho correlation assessment found that PM_2.5_ was not significantly correlated with cognitive outcomes in R1 and R2. However, the correlations suggested that an increase in PM_2.5_ was associated with worse working memory, inhibitory control, nonsymbolic skills, and arithmetic skills, at least in R1. We used each participant’s zip code-aggregated Social Vulnerability Index, which range from 0 to 1, with higher numbers indicating more social vulnerability. Wilcoxon Rank-Sum tests indicated that participants living in higher SVI zip codes (≥0.70; *n* = 15) were not significantly different from those living in lower SVI zip codes (<0.70; *n* = 14), in terms of their PM_2.5_ exposures and cognitive performance in each round. We also found that socioeconomic characteristics mattered, such that children whose parent (s) had at least a Master’s degree or earned more than $100,000 a year had lower PM_2.5_ exposures than children in the other end.

## Introduction

A wealth of research shows that children’s physical environments influence their psychosocial, cognitive, and socio-emotional development (see Evans, 2006; Ferguson et al., 2013 for reviews). When exposed to built environmental factors such as neurotoxic pollutants, noise, crowding, neighborhood poverty, or substandard housing, children exhibit greater negative externalizing behaviors, lower performance in IQ, cognitive, language, and academic tests, and changes in brain structure, attention, social skills, and anxiety, among others (Evans, 2006; Ferguson et al., 2013; Bennett et al., 2016). Recognition of the substantial vulnerability of the nervous system to environmental effects is growing, especially regarding the developing brain (Bennett et al., 2016; National Academies of Sciences, Engineering, and Medicine, 2020). The focus of this study is on the negative impact of air pollution and social vulnerability of the built environment on cognitive function among school-age children.

A growing body of human studies associate exposure to combustion-related air pollutants (PM_2.5_, polycyclic aromatic hydrocarbons, nitrogen dioxide, black carbon) with adverse effects on brain development, including deficits in intelligence, memory, and behavior (Brockmeyer and D’Angiulli, 2016; Clifford et al., 2016; Xu et al., 2016; Payne-Sturges et al., 2019). Inhaled air pollutants deposit into the respiratory tract and migrate to the central nervous system *via* the olfactory epithelium, the blood–brain barrier, or sensory afferents found in the gastrointestinal tract (Kleinman et al., 2008; Block et al., 2012). Alterations in the central nervous system may directly and indirectly affect the brain, such as through the cardiovascular, pulmonary, and immune systems (Block et al., 2012). Prenatal exposures to chemical exposures can, in the long term, negatively affect regions involved in the regulation of emotion, stress, and behavior (Margolis et al., 2022; Peterson et al., 2022) as well as cognitive functioning (Guxens et al., 2018), as measured *via* blood flow, cortical thickness, tissue microstructure, and hippocampal and cerebral volumes, among others. In children, the blood–brain barrier is more permeable during development than later in life, making childhood a period of extreme vulnerability to toxic exposures (Calderón-Garcidueñas et al., 2008). Air pollution exposures may interfere with neural processes, such as neuronal growth and synaptic processes, which are most active during infancy and in childhood, and such interference may affect brain development (Block et al., 2012). Therefore, children who are exposed to higher amounts of pollution may have impaired cognitive performance relative to those who are less exposed (Allen et al., 2017).

In this study, we are interested in cognitive skills researchers have identified as important during middle childhood for later academic success: executive functions and mathematics. Executive functions are a set of top-down skills that are used to perform goal-oriented, effortful tasks (Diamond, 2013) and include working memory and inhibitory control. Working memory helps store and manipulate information mentally, while inhibitory control helps suppress automatic, predominant responses. While these functions develop differentially across the lifespan (Huizinga et al., 2006), executive functioning, in general, at early and middle childhood have been found to be important for cognitive development and academic achievement, including math and reading (Best et al., 2009; Zelazo et al., 2016). In addition to these cognitive skills, foundational mathematical abilities such as non-symbolic skills, which reflects a perception or sense of number (Feigenson et al., 2013), and arithmetic are influential in predicting academic achievement (Schneider et al., 2017) and success in life and in the workplace (Parsons and Bynner, 1997). These cognitive skills are interrelated: working memory and inhibitory control are significant predictors of mathematical achievement throughout elementary school (see meta-analysis by Spiegel et al., 2021), and executive functions and numerical abilities occupy similar brain regions in the prefrontal cortex (Arsalidou et al., 2018).

We examine these effects in the context of the built environment, in which these effects may be exacerbated by local social vulnerability; in such context, individuals may experience a suite of socioenvironmental stressors that lead to increased cumulative risk exposure (e.g., Payne-Sturges et al., 2019). A broad but very important implication of these findings, both in research and practice, is a greater consideration of the environment (and its impact) on children’s cognitive and academic functioning.

### Effects of air pollution on cognitive abilities

PM_2.5_ are inhalable fine particles that are 2.5 micrometers or smaller in diameter and are found in ubiquitous sources such as automobile exhaust (US EPA, 2016). Increased exposure to PM_2.5_ and other pollutants have been associated with neurological effects (Payne-Sturges et al., 2019), lower IQ (Porta et al., 2016), developmental disorders, such as autism (Talbott et al., 2015), and reduced white and gray matter in the brain (Mortamais et al., 2019; Beckwith et al., 2020; Cserbik et al., 2020). Importantly, PM_2.5_ has been associated with poorer performance on specific cognitive tests, such as working memory (Alvarez-Pedrerol et al., 2017; Forns et al., 2017; Rivas et al., 2019; Gui et al., 2020) and inhibitory control (Gui et al., 2020; Margolis et al., 2021), as well as academic assessments (Shier et al., 2019; Mullen et al., 2020). The negative impact of air pollution on cognitive functions is well-documented across the lifespan, including infancy, early and middle childhood, adolescence, and adulthood (Lertxundi et al., 2015; Zhang et al., 2018; Margolis et al., 2021; Miller et al., 2021).

Individual-and context-specific factors, such as age, sex, socioeconomic position, and characteristics of the individuals’ built environment, may not only affect the amount of pollution to which an individual is exposed but also amplify the adverse effects of those exposures (O’Neill et al., 2003; Thomson, 2019). For example, Clougherty et al. (2007) examined the combined effects of exposure to both socio-environmental (violence) and physico-chemical (air pollution) stressors, finding that among children who experienced more than the median scaled violent event exposure, for every standard deviation increase in NO_2_ exposure, there was an associated 1.63 increased odds of asthma; similarly, for children exposed to high stress, there was an increased asthma risk associated with modeled traffic-related pollution exposure (Shankardass et al., 2009). Household income and parent education may indirectly contribute to exposure in PM_2.5_ as they might define household characteristics, such as choices of school and neighborhood (Bell and Ebisu, 2012; Huang et al., 2019). Finally, in a large study of over 10,000 9-10-year-olds, higher residential PM_2.5_ exposure levels were associated with participants who were ethnic minorities (Hispanic or Black) and had parents with a lower level of education and an annual income of less than $49,999 (Cserbik et al., 2020). It is unknown whether these findings are contingent upon the method by which PM_2.5_ exposure is measured. Whereas the aforementioned studies relied upon stationary air sensors, modeled PM_2.5_, and remote sensing to assess PM_2.5_ exposure, none of these studies have used wearable personal air quality monitors that are able to achieve round-the-clock measurement whether the child was outdoors or indoors at a given time.

While a significant literature base has established that air pollution impacts cognitive functioning in children, additional research is needed to understand these relationships. We address several limitations in previous studies, the novel contribution being the use of personal real-time air pollution exposure devices. Previous studies, using longitudinal cohort data, have used aggregate, geographically-matched pollutant exposure as their independent variables, making it difficult to understand the causal pathways that differentially shape poorer neurological outcomes in some children based on their immediate air pollution and social exposure histories. Furthermore, stationary air quality monitors, managed by regulatory entities, cover broad areas and cannot capture instances of the extreme, localized pollution exposure spikes that may be very consequential to children’s neurodevelopment. Additionally, computational models that estimate long-term exposure may also mischaracterize personal exposure. Accurately assessing children’s exposure to air pollution is intrinsically difficult due to the high spatiotemporal variability of combustion-related air pollution, the unique time–activity patterns of children, including time spent indoors at home and school, in vehicles, and walking, bicycling or playing near traffic sources during peak exposure periods (Brauer, 2010; Cattaneo et al., 2010). Therefore, children may experience high peak exposures over short time periods which cannot be captured by stationary air monitoring. Previous studies linking air pollution to children’s development have used a combination of stationary air monitoring and spatial models to estimate long-term exposure. Studies have demonstrated that ambient concentrations and models for air pollutants can mischaracterize personal exposure. This discrepancy is particularly important for children, who are highly susceptible to these exposures due to their ongoing respiratory, cognitive, behavioral and neurological development. Thus, a personal real-time air pollution exposure device may compensate for these limitations and would allow for more correct classification of pollutants and for a more accurate and precise role of air pollution on cognitive and academic outcomes.

### Social vulnerability in the built environment

Though individual-level sociodemographic characteristics are relevant covariates when investigating the relationship between air pollution exposure and cognitive performance, neighborhood characteristics may be adjusted for when modeling the relationship between air pollution (PM10 and ozone) and adult’s health (Chen and Schwartz, 2009). A tool used to assess neighborhood resilience (or, conversely, neighborhood vulnerability) is the Center for Disease Control and Prevention’s Agency of Toxic Substances Data Registry Social Vulnerability Index (SVI), which is a score composed of 15 demographic characteristics for each census tract in the United States using data collected by the US Census and the American Community Survey (Flanagan et al., 2018). This tool’s intended use is for evaluating community vulnerability with regard to disaster preparedness—in recent years, it has been used in environmental health studies to capture the vulnerability of the neighborhood in which study participants live.

In summary, we aim to test the effects of real-time PM_2.5_ exposure on cognitive outcomes, while also accounting for demographic and neighborhood characteristics. Our research questions are:

1. To what extent does air pollution associate with cognitive performance? We hypothesize that average PM_2.5_ air pollution exposure over the time period of data collection is negatively and significantly correlated with cognitive performance.
2. Are there differences in air pollution and cognitive performance over time? Based on trends about PM_2.5_ exposure in the US, participants may be exposed to higher concentrations in the months of January and July (i.e., Winter and summer), and lower concentrations in the late March and mid-October (Zhao et al., 2018).
3. Do demographic and neighborhood characteristics relate to air pollution exposure and cognitive outcomes? Children whose parents have higher levels of education, minority (non-White) status, and greater incomes may be exposed to lower amounts of air pollution compared to their peers (Cserbik et al., 2020). These children may also have better performance in cognitive and academic assessments (Koponen et al., 2007; Dahl and Lochner, 2012; Last et al., 2018).

## Materials and methods

This research was designed as a pilot study to investigate the cognitive effects of short-term PM_2.5_ exposures among children ages 7 to 11 years residing in the Washington D.C. metropolitan area. We were interested in how variations in day-to-day exposure to air pollution impact children’s cognitive performance on mathematical tasks designed to assess the underlying cognitive processes relevant to numerical cognition. We aimed to conduct the air pollutant exposure assessment campaign across different seasons, specifically the warm and cold periods of the year and measure children’s short-term personal exposures to PM_2.5_ using *Flow*, a small, portable device that could be worn or attached to a backpack.

### Recruitment strategy and participant selection

Our initial sample consisted of 30 children ages 7 to 11 years recruited *via* a university-run infant and child database, social media postings, and bulletins posted on parks, shopping malls, and other public areas. We excluded children if they had asthma and/or lived in a house where smoking occurred within the home. Recruitment occurred in a large metropolitan area with more than six million people and a median household income of more than $106,000. Additionally, 51% of households in the area had married couples, 51.7% had a Bachelor’s degree or more.^1^ The race breakdown in the area was 51.9% White, 25.2% Black or African American alone, 10.4% Asian, 7% another single race alone, and 5.6% two or more races.

Twenty-nine children and their parents/guardians completed two rounds, between January 2020 and August 2021, with most of the participation occurring the COVID-19 pandemic. In Round 1, 28 out of 30 participants completed the procedure between October 2020 and February 2021 (the two other participants completed the procedure between January and March 2020). In Round 2, 27 participants completed the procedure between May and August 2021 (one in October 2020). One participant from Round 1 did not participate in Round 2.

The sample of participants with complete data is *n* = 21. Table 1 provides the distribution of both initial and complete sample by parental marital status, education, and household income as well as by children’s race, age, and ethnicity, as reported by the parent who completed the baseline demographic survey.

**Table 1:** Demographic breakdown for the full sample (*N* = 30) and the sample with complete data (*n* = 21).

	Initial sample		Complete sample	
Characteristic				
Child’s sex				
Male	14	46.67%	10	47.62%
Female	16	53.33%	11	52.38%
Parent’s marriage status				
Married	27	90.00%	19	90.48%
Never Married	2	6.67%	1	4.76%
N/A	1	3.33%	1	4.76%
Child’s race				
White	20	66.67%	16	76.19%
Black or African-American	3	10.00%	1	4.76%
Asian	3	10.00%	2	9.52%
Multi-racial, or n/a	4	13.33%	2	9.52%
Child’s ethnicity				
Non-Hispanic/Latinx	25	83.33%	19	90.48%
Hispanic/Latinx	4	13.33%	2	9.52%
Age				
7	4	13.33%	2	9.52%
8	10	33.33%	8	38.10%
9	8	26.67%	6	28.57%
10	7	23.33%	5	23.81%
11	1	3.33%		
Annual household income				
Between 20,000-50,000	2	6.67%	2	9.52%
Between 50,000-100,000	4	13.33%	2	9.52%
Above 100,000	23	76.67%	17	80.95%
Parent education				
Some college	2	6.67%	1	4.76%
Bachelor’s	10	33.33%	7	33.33%
Master’s and/or Professional Degree	18	60.00%	13	61.90%
SVI				
Greater than 0.70	8	26.67%	7	33.33%
Less than 0.70	22	73.33%	14	66.67%

Complete sample consists of participants who had a complete data set.

### Informed consent procedures

This study was approved by the University of Maryland-College Park Institutional Review Board. As this study’s launch coincided with the beginning of the COVID-19 pandemic lockdown, we submitted a revised protocol in accordance with university guidelines regarding the conduct of research during the COVID-19 pandemic. We developed a contactless drop-off/pick-up procedure for the safe distribution of equipment needed for study participation. Parent participant consent and child assent procedure are described below.

### Procedure

**Figure 1:**
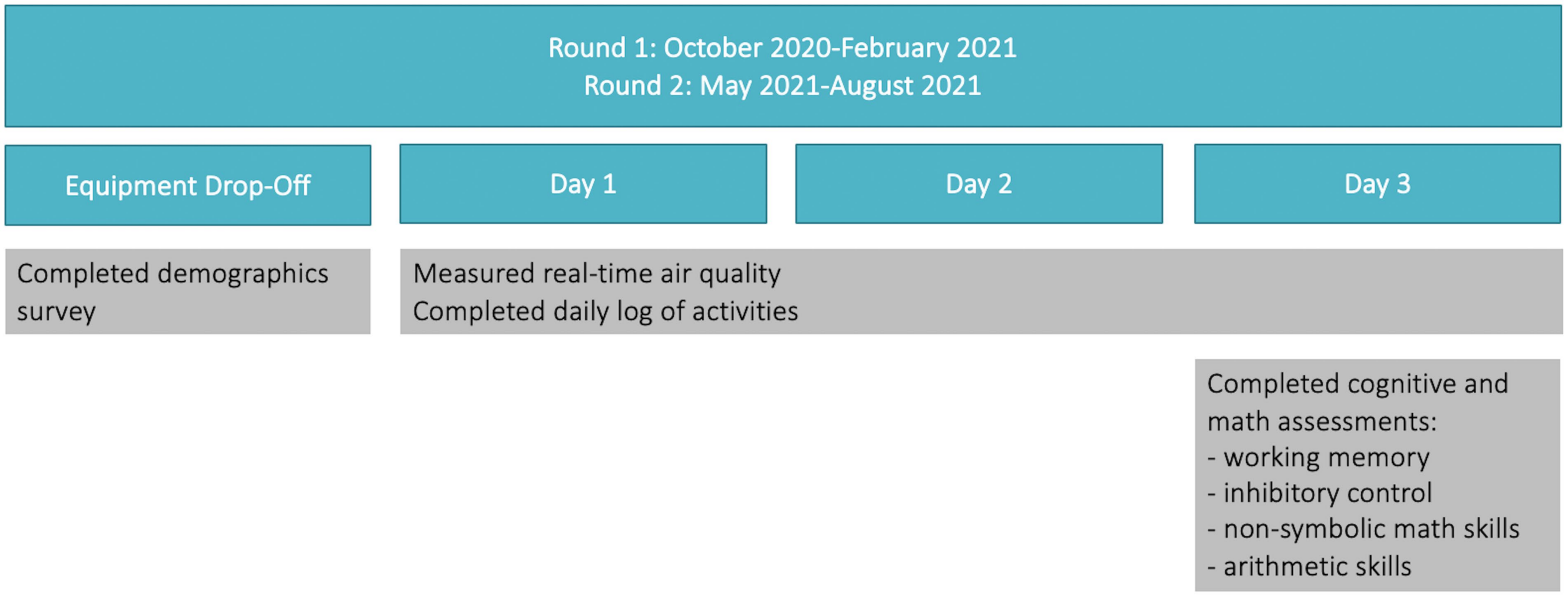
Procedure. Participants completed two rounds. Each round had the same procedure. At equipment drop-off, parents completed a demographics survey, after which the child participants wore the Flow device for 3 days. At Day 3, they completed cognitive and math assessments.

Figure 1 shows the study procedure. Children completed two rounds of the study, and each round consisted of the same procedure of 3 days of PM_2.5_ exposure monitoring followed by cognitive assessments. On the first day, study personnel delivered the study kit to participants homes. Each participating family received a 13 in. × 8 in. × 4 in. plastic box in which we placed a Flow Air Quality sensor, a Samsung tablet with which the sensors was synced for data collection, Mi-Fi devices to provide the Wi-Fi needed to support these devices and the associated chargers with each device and a laminated series of instructions for use, The tablet was also used for the parents’ consent forms, children’s’ assent forms and baseline survey, all of which were combined into one survey hosted on Qualtrics. Children’s daily activities were also noted in a Qualtrics log that was completed each of the 3 days of participation. Also on the Qualtrics platform, the cognitive assessments that children completed at the conclusion of participation were also available on the tablet. During each of the two rounds of participation, this package was dropped off by the research team at participants’ homes.

In the first round, the parent completed the consent form and a questionnaire about their household (including demographics) initially, while in the second round, they were asked to provide any updates on household information. Children completed assent forms. After completing the survey, participants were asked to ascertain the connection between the Flow air quality sensor (see next sub-section) and the Samsung tablet. Children wore the air quality sensor on a lanyard provided by the study team over the course of their participation. Children were instructed to keep the sensor by their beds while sleeping. At the end of each day, parents logged their children’s activities using a Qualtrics form, which allowed parents to enter a date and identify an activity from a drop-down menu (e.g., Indoors at home, Outdoors at school, etc.) for each 15-min period in a day covering 3 days of the exposure assessment. On the second day, researchers sent an email or text message reminder to parents to remind their children to complete the cognitive tests the following day on the tablets before picking up the equipment. Study personnel communicated with the families by email or text messaging to remind them of the procedures and to check if they had any concerns. On the third day, researchers picked up the equipment after children completed their cognitive tests available on the tablet and their last period of Flow monitoring.

### Air pollution personal exposure assessment

We used low cost (~$150/monitor) and commercially available sensors, Flow air quality sensor, from Plume Labs^2^ to measure real-time ambient air quality. The Flow sensor measured pollutants (NO_2_, VOCs, PM_10_ and PM_2.5_) in the ambient air by air being drawn into the device through holes drilled into the body of the device with a small mechanical fan. Particulate matter was measured as the amount of laser-produced light diffracted by particles, and the sensors produced reports of ambient air quality every minute. Calibration of these tools within this sensors is executed *via* machine learning processes enabled through internet connection to the sensor.^3^ The accuracy, precision and overall performance of Flow sensors has been investigated by and reported in Crnosija et al. (2022), which found that these sensors are able to detect changes in ambient PM_2.5_ and PM10 reliably. Specifically, a coefficient of determination of 0.76 (*R*^2^ = 0.76) was obtained for the relationship between minute-by-minute PM_2.5_ exposure in 32 Flow devices and a Plantower air sensor, indicating how well the average PM_2.5_ measured by the Flow devices predicts that measured by the Plantower air sensor (Crnosija et al., 2022).

For the device to be properly calibrated and collect time-stamped sensor air quality and spatial data, Wi-Fi and GPS connectivity with a companion device (i.e., a Samsung Tablet) were also required. To ensure that Wi-Fi connectivity was maintained throughout a subject’s possession of the device, each family was given a Tablet paired with the Flow device and a Mi-Fi, a small Wi-Fi hotspot that provided internet connectivity to the Flow device and the Tablet. Though the Tablet and Mi-Fi were left at home during the child’s day, the family would make sure that connectivity among all devices was made upon the child’s return home.

The PM_2.5_ data was downloaded from the sensors and time stamps were converted from UTC to EST, accounting for season. Then time-stamped data was aggregated into 15-min increments; this process allowed us to generate averages of PM_2.5_ exposure for every 15 min data was collected. These 15-min averages were then matched to the days of Time Activity Logs, wherein parents used a Qualtrics interface to identify their child’s activity at a given time. This coding was completed using Python 3.

### Social vulnerability index

Each family was assigned a SVI (a value between 0.0 and 1.0) based on their zip code, with higher values suggesting greater risk of social vulnerability. The value is based on the index provided by the CDC, specifically the overall tract summary ranking variable. Census tracts within the state of Maryland were given an SVI value ranging from 0.000 to 1.000, with 1.000 representing the 100^th^ percentile for extreme social vulnerability and 0.000 representing the 0^th^ percentile for the lowest level social vulnerability, ranked against one another. We use the SVI as a proxy to capture neighborhood effects and the local built environment. We downloaded the 2018 SVI .csv file for Maryland from the CDC SVI web portal and merged it with a tract-ZIP code “crosswalk” file provided by the Department of Housing and Urban Development to “translate” census tracts to ZIP code areas. These two pieces of data were spatially merged using the merge command in Python; the resultant dataset was then exported in an .csv format. As there were multiple entries for each zip code, these values were aggregated by finding the mean by each zip code in the dataset. This dataset was then exported in .csv format and then converted to .xlsx format for merging to the master dataset in Stata.

From this continuous SVI variable, a dichotomous variable was created to categorize participants based on a cutoff of 0.70, representing the upper bound of the scale, with participants with SVI value of more than or equal to 0.70 being coded as 1 (high vulnerability) and those with an SVI of less than 0.70 as 0 (low vulnerability).

### Cognitive measures

**Figure 2:**
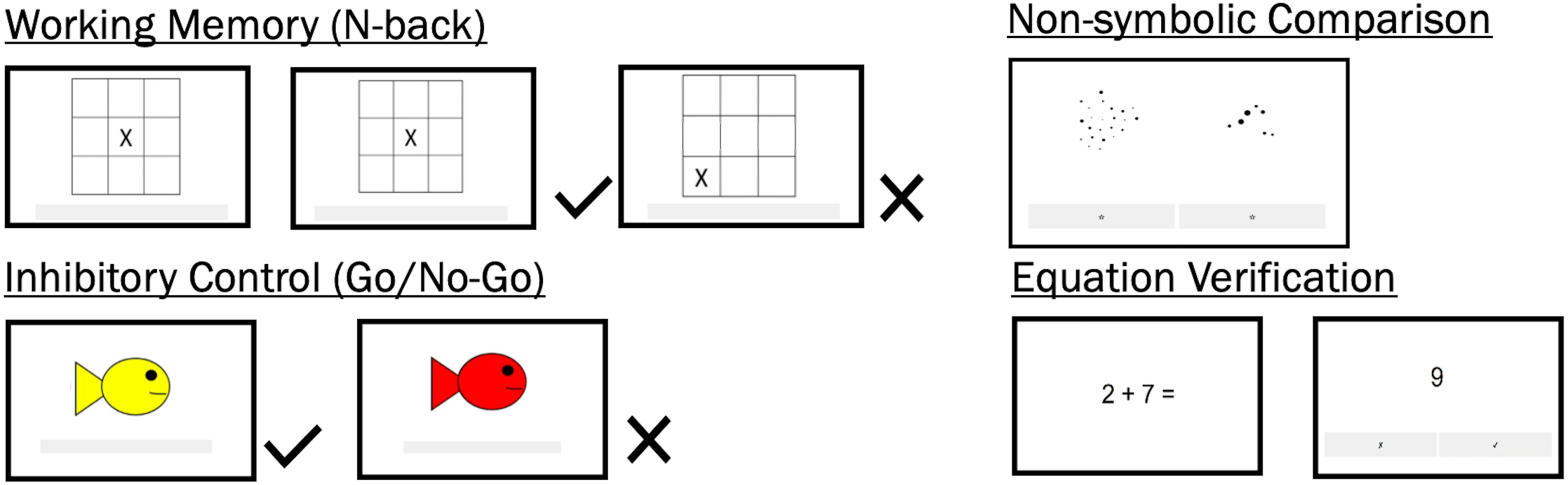
Cognitive Tasks. Participants completed four cognitive tasks. See Methods section for the tasks’ description.

The child participants completed tasks that measured their working memory (Kane et al., 2007; Antonini et al., 2013), inhibitory control (Kim et al., 2007), non-symbolic comparison ability (Gebuis and Reynvoet, 2012a, b), and arithmetic ability (Jasinski and Coch, 2012). We refer to all tasks collectively as cognitive tasks. The tasks were completed on Qualtrics on a Samsung tablet provided by the research team. Split-half reliabilities were calculated for each block of each task by correlating the first half of the trials for each block to the second half. Figure 2 shows example trials from each task.

#### Working memory: N-back task

Children were shown a grid of nine 3×3 cm cells, with an ‘X’ in one of the cells. They are told to press a button if a trial is similar to the previous one (target; i.e., the ‘X’ did not move) and to not press anything if a trial is dissimilar to the previous one (non-target). Participants were presented with 10 practice trials with feedback and 160 experimental trials, distributed over 2 blocks, without feedback. Each block had 24 targets (i.e., the trial is similar to the previous one) and 56 non-targets, with an option to rest between blocks. Each trial was shown for 1,000 and 1,250 milliseconds, with 500–1,000 millisecond pause between trials. A trial was a “hit” if a participant correctly pressed a target; a trial was a “false alarm” when the participant pressed a non-target. For each participant, a d’ statistic, which accounted for hits and false alarms, was recorded. Higher values of d’ indicated better working memory. Split-half reliability coefficients in Round 1 were r(29) = 0.84 for Block 1 and r(29) = 0.66 for Block 2. Split-half reliability coefficients in Round 2 were r(23) = 0.96 for Block 1 and r(23) = 0.92 for Block 2.

#### Inhibitory control: Go/No-Go task

Children were shown two types of stimuli, a red or a yellow fish, and were told to press a button when encountering a yellow fish (the Go stimulus) and to not press anything when encountering a red fish (No-Go). Participants were presented with 10 practice trials with feedback and 280 experimental trials, distributed over 4 blocks, without feedback. Each block had 42 Go trials and 14 No-Go trials, with an option to rest between blocks. Each trial was shown for between 1,000 and 1,250 ms, with 500–1,000 ms pause between trials. For each participant, the number of commission errors (i.e., pressed the button when No-Go stimulus appeared) and commission error rate (i.e., number of commission errors divided by 56) were recorded. The commission error rates were then reversed (1 minus commission error rate), so that higher values indicated better inhibitory control. Split-half reliability coefficients in Round 1 were *r*(29) = 0.88 for Block 1, *r*(29) = 0.79 for Block 2, *r*(29) = 0.51 for Block 3, and *r*(29) = 0.87 for Block 4. Split-half reliabilities in Round 2 were *r*(23) = 0.26 for Block 1, *r*(23) = 0.33 for Block 2, *r*(23) = 0.61 for Block 3, and *r*(23) = 0.83 for Block 4.

#### Non-symbolic comparison task

Children saw two sets of dots on the screen and were asked to choose which set has more dots. There were 20 unique pairs of dots in total, and these pairs were displayed with varying sizes, such that sometimes, the more numerous have bigger dots (congruent) and sometimes the less numerous have bigger dots (incongruent). There were 4 blocks of 20 trials each; Blocks 1 and 4 were fully congruent; Blocks 2 and 3 were fully incongruent. Participants completed 6 practice trials that were not analyzed. Participants’ accuracy was recorded. Split-half reliability coefficients were *r*(29) = 0.81 for Block 1, *r*(29) = 0.85 for Block 2, *r*(29) = 0.65 for Block 3, and *r*(29) = 0.45 for Block 4 in Round 1. Split-half reliability coefficients in Round 2 were *r*(23) = 0.41 for Block 1, *r*(23) = 0.47 for Block 2, *r*(23) = 0.30 for Block 3, and *r*(23) = 0.52 for Block 4.

#### Equation verification task

Children were shown a single-digit addition equation in two screens, such as that the problem (e.g., ‘2×2=’) is followed by its answer (e.g., ‘4’). They are told to press a button if the answer was right and to not press anything when the answer is wrong. 112 equations were created, such that 56 problems were presented twice with either the right or a wrong answer. The 56 problems had nonidentical addends and the wrong answer was either 1 or 3 more than the right answer. Participants were first shown practice trials, after which the equations were randomized and shown across 2 experimental blocks. Trials began with the presentation of a warning signal (‘+’) in the center of the screen for a duration of between 500 and 1,000 ms, after which the problem appeared for 1,500 ms, followed by a correct or wrong answer for 1,500 ms and a blank screen for 1,500 ms. Participants were allowed to respond as soon as they saw the answer, so they had 3,000 ms to respond. Participants’ accuracy was recorded. Split-half reliability coefficients in Round 1 were *r*(29) = 0.80 for Block 1 and *r*(29) = 0.66 for Block 2. Split-half reliability coefficients in Round 2 were *r*(23) = 0.84 for Block 1 and *r*(23) = 0.71 for Block 2.

### Data preparation

The final dataset consisted of all 30 participants’ demographic data, dichotomized SVI values, PM_2.5_ air pollution exposure over 3 days on each round, and performance in the cognitive tasks on each round.

## Results

We present our results according to our research questions. Our first two questions explore the extent to which air pollution exposure associate with cognitive performance (Research Question #1) and whether there are differences in air pollution exposure and cognitive performance over time (Research Question #2). We present descriptive statistics, as well as results from Spearman’s rho correlation and quantile regression analyses. Our last question concerns whether air pollution exposure and performance in cognitive tasks differ by demographic characteristics, such as sex, income, parental education, and dichotomized SVI (Research Question #3). We present results of Wilcoxon rank-sum tests. Due to the small sample size, we could not infer the distribution of the data and therefore used nonparametric tests for our analysis. Analysis was conducted in Stata 14 (College Station, TX).

### Description of PM_2.5_ exposure and outcomes

We first describe round-by-round data and then proceed to examine complete data across both rounds. Table 2 lists the descriptive statistics for the PM_2.5_ and cognitive measures in Round 1 (*n* = 28) and Round 2 (*n* = 23). Average 3-day PM_2.5_ exposures were below the EPA standard in both Round 1 [*Median* = 3.66, *Mean (M)* = 6.22, *standard deviation (SD)* = 10.32] and Round 2 (*Median* = 3.01, *M* = 3.13, *SD* = 1.29). There was an outlier in Round 1 (i.e., greater than an absolute *z*-score of 3), but we kept that data point in our analysis.

**Table 2:** Descriptive statistics in Round 1 (*n* = 28) and Round 2 (*n* = 23).

	Round 1		Round 2	
	Mean	SD	Mean	SD
Flow 3-day Average	6.22	10.32	3.13	1.29
N-back d’	0	1.71	0	1.68
Inhibitory Control Commission Errors	4.36	4.75	2.30	2.62
Dot Comparison	0.64	0.17	0.73	0.13
Equation Verification acc	0.88	0.11	0.91	0.09

*N* in each round indicates the number of participants that had full data in the given round.

Table 3 lists the descriptive statistics for those with complete data (*n* = 21). Similar patterns emerged. Compared to Round 1, these children committed fewer errors in the inhibitory control task (3.05 vs. 2.05) and were more accurate in the non-symbolic (64% vs. 73%) and arithmetic tasks in Round 2 (91% vs. 92%). Wilcoxon signed-rank tests were used to determine whether there were differences between Round 1 and Round 2. The tests revealed a near-significant difference in three-day average PM_2.5_ readings between Round 1 and Round 2 (*Z* = −1.96; *p* = 0.05), and no differences in three cognitive measures: working memory (*Z* = −1.13; *p* = 0.26), inhibitory control (*Z* = −1.52; *p* = 0.10), and arithmetic (*Z* = −0.87; *p* = 0.39). The only significant difference from Round 1 to Round 2 is in non-symbolic comparison skills (*Z* = −2.24; *p* = 0.02).

**Table 3:** Descriptive statistics and Spearman’s rho correlation coefficients for participants with complete data (*n* = 21).

	Round 1		Round 2			1	2	3	4	5
	*M*	*SD*	*M*	*SD*	*p*					
1. Flow 3-day Average	7.06	11.81	3.06	1.29	0.05		0.25	0.18	−0.11	0.17
2. Working Memory d’	0.34	1.57	−0.04	1.75	0.26	−0.30		0.21	0.46^*^	0.53^*^
3. Inhibitory Control Commission Errors ^a^	3.05	3.11	2.05	2.27	0.13	−0.21	0.55^**^		0.25	0.11
4. Non-Symbolic Comparison	0.64	0.20	0.73	0.13	0.02	−0.11	0.34	0.29		0.50^*^
5. Arithmetic	0.91	0.07	0.92	0.07	0.39	0.06	0.51^*^	0.49^*^	0.05	

Statistical tests consisted only of participants who had full data for both rounds. Round 1 on lower triangle and Round 2 on upper triangle. *p* values are from Wilcoxon signed rank tests of performance between Round 1 and Round 2.

a For correlational analysis, we used 1-commission error rate.

* *p* < 0.05 and

** *p* < 0.01.

### Associations between exposure and outcomes

Table 3 also shows the Spearman’s Rho correlations between PM_2.5_ and cognitive measures for those who had completed data across both rounds (*n* = 21). The correlations suggested that cognitive measures (working memory and inhibitory control) were correlated with mathematical outcomes. In Round 1, working memory was correlated with arithmetic performance (*r_s_* = 0.81, *p* = 0.02). Inhibitory control was also correlated with arithmetic performance (*r_s_* = −0.49, *p* = 0.02). In Round 2, working memory was correlated with non-symbolic comparison skill (*r_s_* = 0.46, *p* = 0.04) and arithmetic performance (*r_s_* = 0.50, *p* = 0.01). Non-symbolic comparison skill was significantly correlated with arithmetic (*r_s_* = 0.50, *p* = 0.02).

We conducted quantile regressions to examine whether the associations between PM_2.5_ exposure and children’s cognitive outcomes depend on the latter. Quantile regression is a relatively new approach that allows for examination at extreme values of an outcome variable (Koenker, 2005). In separate quantile regressions, we explored the possibility that the PM_2.5_ exposure may affect cognitive outcome at lower quantiles of outcome performance. We started by standardizing all variables (except working memory, which was already standardized) and then dividing the outcomes into deciles. Then, each outcome was regressed to PM_2.5_ exposure in each round using the quantreg package in R (Koenker, 2005).

Table 4 lists the regression coefficients. In Round 1, all regression coefficient estimates were negative at the lowest four percentiles, indicating that at the lowest quantiles (i.e., children who performed poorly), children showed negative associations between outcomes and PM_2.5_ exposure. However, the only significant association was for inhibitory control at the 40th percentile, with a coefficient of −0.34, .95 *CI*[−0.95, −0.03]. In Round 2, the patterns were different. For working memory, the lowest quantiles had high, positive coefficients; for inhibitory control, the coefficient of almost every quantile was zero. There was no discriminable pattern for non-symbolic and arithmetic skills and no coefficients were significant.

**Table 4:** Quantile regression coefficient estimates and 95% confidence intervals with exposure as predictor.

Rnd	Qtle	Working memory	Inhibitory control	Non-symbolic comparison	Arithmetic
1	0.10	−0.15 [−3.83, 0.53]	0.56 [−8.77, 0.57]	−0.15 [−5.35, 0.47]	−0.23 [−2.71, 0.10]
	0.20	−0.53 [−1.05, 0.36]	−0.23 [−1.56, 0.14]	−0.43 [−1.21, 0.37]	−0.06 [−2.03, 0.45]
	0.30	−0.58 [−1.31, 0.25]	−0.24 [−0.96, 0.20]	−0.30 [−0.90, 0.06]	−0.27 [−1.42, 0.38]
	0.40	−0.51 [−0.95, 0.10]	−0.34 [−0.95, −0.03]	−0.17 [−0.64, 0.05]	−0.33 [−0.54, 0.23]
	0.50	0.02 [−0.86, 0.27]	−0.37 [−0.61, 0.03]	−0.03 [−0.50, 0.02]	−0.29 [−0.42, 0.21]
	0.60	0.09 [−0.78, 0.29]	−0.10 [−0.48, 0.16]	−0.04 [−0.25, 0.52]	−0.11 [−0.45, 0.14]
	0.70	−0.04 [−0.83, 0.53]	−0.12 [−0.52, 0.57]	−0.12 [−0.14, 0.60]	0 [−0.48, 0.10]
	0.80	−0.08 [−0.18, 0.72]	−0.15 [−0.33, 0.53]	−0.17 [−0.20, 0.97]	0.06 [−0.21, 0.22]
	0.90	−0.12 [−0.12, 1.60]	−0.18 [−0.19, 5.45]	0 [−0.26, 1.74]	0 [0, 1.45]
2	0.10	0.39 [−0.06, 0.82]	0 [−0.47, 0.50]	−0.88 [−1.44, 0.63]	−0.12 [−0.73, 0.39]
	0.20	0.46 [−0.39, 0.79]	0.29 [−0.65, 0.61]	−0.15 [−1.05, 0.11]	0.20 [−1.26, 0.29]
	0.30	0.20 [−0.17, 0.92]	0 [−0.79, 0.41]	−0.10 [−0.48, 0.07]	0 [−0.97, 0.50]
	0.40	0.10 [−0.30, 0.64]	0 [−0.29, 0.13]	−0.16 [−0.48, 0.01]	−0.16 [−0.50, 0.36]
	0.50	−0.09 [−0.21, 0.64]	0 [−0.29, 0.19]	−0.26 [−0.51, 0.14]	0 [−0.26, 0.68]
	0.60	−0.01 [−0.18, 0.49]	0 [−0.34, 0.26]	−0.30 [−0.61, 0.26]	0 [−0.28, 0.70]
	0.70	0.03 [−0.21, 0.24]	0 [−0.13, 0.21]	−0.40 [−0.63, 0.33]	−0.08 [−0.34, 0.38]
	0.80	0.03 [−0.11, 0.17]	0 [−0.15, 0.44]	−0.40 [−0.54, 0.35]	0.05 [−0.46, 0.47]
	0.90	0 [−0.21, 0.12]	−0.13 [−0.15, 10.05]	0.11 [−0.61, 0.90]	0.05 [−0.50, 0.23]

### The role of demographic and neighborhood characteristics

To tease out the associations and account for covariates, we conducted separate Wilcoxon rank-sum tests by round and by demographic variable (sex, income, parental education, and SVI). Table 5 consists of these statistics. For Round 1 (*n* = 28), there were no significant differences by sex, income, parental education. For Round 2, we found some significant differences: by income and parental education. Children from households who made above $100,000 (*n* = 18) had significantly less PM_2.5_ exposure than children from households who made below 100,000 (*n* = 5), with 2.69 compared to 4.79 (*Z* = −3.09; *p* = 0.002). Additionally, those with parents that had master’s or professional degree (*n* = 9) had significantly less PM_2.5_ exposure (*n* = 14) than those with parents who only finished some or all of college (*n* = 9), with 2.51 compared to 4.09 (*Z* = −3.09; *p* = 0.002). (These results held when for those who only had complete data.) We did not find any other significant differences in other outcome measures or in any demographic category. When comparing those who were assigned an SVI of less than 0.70 and those with SVI of greater than 0.70, we found no differences in PM_2.5_ exposures or the cognitive outcomes in both Round 1 (*p*s: 0.37 to 1) and Round 2 (*p*s: 0.09 to 0.95).

**Table 5:** Differences between demographic categories on all measures.

	PM_2.5_ (3-day ave.)^1^	Working memory d’	Inhibitory control^2^	Non-symbolic comparison^3^	Arithmetic^3^
Sex - Round 1					
Female (*n* = 15)	7.24	−0.208	0.949	0.627	0.849
Male (*n* = 13)	5.04	0.244	0.921	0.658	0.921
Wilcoxon Test *Z*	−0.36	−1.04	−0.67	−0.95	−1.78
*p* value	0.72	0.3	0.5	0.34	0.075
Sex - Round 2					
Female (*n* = 12)	3.4	−0.137	0.96	0.72	0.88
Male (*n* = 11)	2.84	0.149	0.98	0.74	0.95
Wilcoxon Test *Z*	−0.76	−0.40	−1.06	−1.23	−1.81
*p* value	0.45	0.69	0.29	0.22	0.07
Income - Round 1					
Below 100,000 (*n* = 5)	4.34	0.92	0.97	0.67	0.9
Above 100,000 (*n* = 23)	6.63	−0.2	0.93	0.63	0.88
Wilcoxon Test *Z*	−0.23	−0.99	−1.17	−1.17	−0.33
*p* value	0.82	0.32	0.24	0.24	0.74
Income - Round 2					
Below 100,000 (*n* = 5)	4.72	0.2	0.96	0.69	0.87
Above 100,000 (*n* = 18)	2.69	−0.05	0.97	0.74	0.92
Wilcoxon Test *Z*	−3.09	−0.23	−0.28	−0.33	−0.60
*p* value	0.002	0.82	0.78	0.74	0.55
P Education - Round 1					
Bachelor’s or below (*n* = 11)	5.31	0.57	0.95	0.63	0.91
Master’s or Professional Degree (*n* = 17)	6.81	−0.37	0.93	0.65	0.87
Wilcoxon Test *Z*	−3.09	−0.23	−0.28	−0.33	−0.60
*p* value	0.21	0.51	0.35	0.89	0.24
P Education - Round 2					
Bachelor’s or below (*n* = 9)	4.09	0.39	0.97	0.69	0.92
Master’s or Professional Degree (*n* = 14)	2.51	−0.25	0.96	0.76	0.91
Wilcoxon Test *Z*	−3.09	−0.23	−0.28	−0.33	−0.60
*p* value	0.002	0.73	0.32	0.51	0.47
SVI - Round 1					
Less than 0.70 (*n* = 20)	4.21	0.1	0.93	0.63	0.87
Greater than 0.70 (*n* = 8)	11.2	−0.24	0.94	0.66	0.91
Wilcoxon Test *Z*	−3.09	−0.23	−0.28	−0.33	−0.60
*p* value	0.38	0.96	0.98	1	0.57
SVI - Round 2					
Less than 0.70 (*n* = 16)	3.2	−0.03	0.97	0.73	0.9
Greater than 0.70 (*n* = 7)	2.96	0.06	0.96	0.73	0.93
Wilcoxon Test *Z*	−3.09	−0.23	−0.28	−0.33	−0.60
*p* value	0.77	0.95	0.09	0.57	0.81

^1^Median; ^2^1 – commission rate; ^3^accuracy.

## Discussion

In this exploratory study, we sought to examine the associations between children’s personal exposure to PM_2.5_, a common air pollutant, and their cognitive abilities, namely working memory, inhibitory control, non-symbolic skills, and arithmetic ability. Our novel contribution to this literature is our real-time measurement of pollution children encounter on a daily basis in their immediate environments. Our findings also mirror extant findings in the extant literature. We highlight three of them.

First, on average, the children in our sample were exposed to PM_2.5_ below the US EPA National Ambient Air Quality Standard (NAAQS). Though it may be perceived that children who experience exposure less than the NAAQS levels are “safe” from the harmful effects of PM2.5, there are several limitations to reliance upon this standard when evaluating these air pollutant exposures with regard to neurocognitive outcomes. The annual standard fails to capture the effects of brief periods of high exposure, or the effects of indoor air pollution, which fall outside of the purview of the EPA. Further, these PM_2.5_ standards may not provide effective protection against poor neurocognitive health outcomes. We did not have any hypothesis regarding the sample, although we did expect relatively consistent PM_2.5_, given the restricted spatial coverage of our study catchment area, and for the average exposure to be elevated, due to the sample’s proximity to a major metropolitan area (as reported by the EPA’s EJSCREEN tool).

However, there was a nearly statistically significant decrease in exposure (and a significant improvement in non-symbolic comparison ability) between the first and second round of data collection. Two important factors were the variation in schooling in response to the COVID-19 pandemic and the season in which data collection occurred. For a majority of the children (28 out of 30), the first round occurred between October 2020 and February 2021; the second round (for 28 out of 29 children) occurred between May and August 2021.

Second, children’s PM_2.5_ exposure was negatively related to cognitive abilities, though non-significantly. The negative associations correspond to other findings, at least with regard to working memory and inhibitory control (Freire et al., 2010). The current study was the first to include tasks that measured foundational mathematical abilities and found negative associations. However, we found significant associations only in Round 1 (though there was a negative correlation in Round 2). One possible explanation is participants’ near-ceiling improvement from Round 1 to Round 2. Another possible explanation is the time of data collection—Round 1 occurred when a majority of schools in the state were in lockdown (and in the winter), while Round 2 occurred when children were going back to schools in-person. Through the quantile regression analysis, we also found the negative associations between PM_2.5_ and cognitive abilities more for those who did poorly on the cognitive tasks. This suggests that participants at the lower end of cognitive outcome distribution represent a vulnerable group, which is consistent with other studies (e.g., Ebisu and Bell, 2012). Given that air pollution exposure as early as before birth influences children at middle childhood and beyond, future research should continue to study its implications for cognitive and academic outcomes across the lifespan.

Third, there were significant differences in exposure by household income and by parent education, with lower PM_2.5_ exposure for children who were in higher household income and had parent(s) with higher education greater than a Bachelor’s degree, consistent with other findings (Cserbik et al., 2020). These were important variables to consider because these describe social vulnerabilities and may help earners and their dependents cope with environmental hazards (Cutter et al., 2006). While we did not find any significant differences between participants with SVI greater than 0.70 and those with less than 0.70, it is likely that only some components of the index were more important when considering the effects of air pollution, although more research should be done using the widely-used index.

### Limitations and future directions

We were not able to look at whether air pollutant exposure causally affects children’s cognitive abilities. However, a candidate causal pathway for socioeconomic differences in neurocognitive development is exposure to environmental contaminants such as air pollution. Racial, ethnic and socioeconomic differences in air pollutant exposures are well documented (Miranda et al., 2011; Tessum et al., 2019; Rubio et al., 2021), and this may be reflected in the brain. For example, Miller et al. (2021) tracked early life stress experiences of 9–13 year olds for two years, as well as volume changes in specific areas of their brains and their exposure to PM_2.5_ in the participants’ residential areas. They found a stronger negative effect of PM_2.5_ on brain development for adolescents who had less severe early life experiences. Though in contrast to most studies on respiratory health, it demonstrates that at the neural level, there is an interaction of PM_2.5_ exposure, demographics, and life experiences.

Another limitation is the convenience sampling and the small sample size, as the study was exploratory. Future studies should use a sample representative of families at least in the metropolitan area and should continue to explore the role of demographic factors along with air pollution exposures and other variables that measure the built environment, such as noise pollution (Thompson et al., 2022). Currently, it is unclear whether some factors, such as household income and parent education, are more important than others, such as whether the family lives in a more urban than suburban area. It could be that the air pollution is a mediator of these relations: families with certain demographic characteristics are more likely to live in more polluted areas, and therefore, children in those families have lower cognitive or academic attainment.

Relatedly, increasing the sample size to increase statistical power to identify differences among groups and correlation between measures and providing more research support to participants may be beneficial and may help resolve some of the incomplete data. The incomplete data we had were more due to lack of cognitive scores in some of the participants. Participants completed all measures in a tablet, most of them in one sitting. The estimated time of completion was 1 h and 30 min. While families were given explicit instructions regarding how to complete the tests, researchers can provide more guidance, in the form of proctoring, in the future. It is important to note that the measures are more often used in a laboratory setting.

Prospective researchers may look at other air pollutants, such as PAH, NO_2_, and CO_2_, and the reliability of the Flow sensor. Future studies may also compare the measurements from the Flow sensor to stationary monitors, as well as measurements in children’s exposure when at home in comparison to time when not at home. Implications for practice include using such devices in science classrooms, which may not only teach children about air pollution and its impacts but also encourage actions toward reducing air pollution (Varaden et al., 2021).

## Conclusion

The present pilot study aimed to provide evidence for the role of the environment in child development. The results of the study revealed that 1) children’s cumulative exposure to particulate matter, PM_2.5_, over 3 days is negatively related to their cognitive abilities, 2) these relations matter greatly for those with poorer cognitive abilities, and 3) socioeconomic characteristics matter.

## Data availability statement

The raw data supporting the conclusions of this article will be made available by the authors, without undue reservation.

## Ethics statement

The studies involving human participants were reviewed and approved by Institutional Review Board of the University of Maryland College Park. Written informed consent to participate in this study was provided by the participants’ legal guardian/next of kin.

## Author contributions

DP-S and RP planned this study. NC and JM collected the data, under the supervision of DP-S and RP. JM analyzed the data, with support from NC, who also constructed the project datasets. All authors contributed to the article and approved the submitted version.

## Funding

This research was supported by the University of Maryland Catalyst Fund.

## Conflict of interest

The authors declare that the research was conducted in the absence of any commercial or financial relationships that could be construed as a potential conflict of interest.

## Publisher’s note

All claims expressed in this article are solely those of the authors and do not necessarily represent those of their affiliated organizations, or those of the publisher, the editors and the reviewers. Any product that may be evaluated in this article, or claim that may be made by its manufacturer, is not guaranteed or endorsed by the publisher.

## Footnotes

^1^ www.censusreporter.org

^2^ www.plumelabs.com

^3^ https://plumelabs.zendesk.com/hc/en-us/articles/360009014973-How-does-Flow-work-
